# Ensemble Analysis of Angiogenic Growth in Three-Dimensional Microfluidic Cell Cultures

**DOI:** 10.1371/journal.pone.0037333

**Published:** 2012-05-25

**Authors:** Waleed A. Farahat, Levi B. Wood, Ioannis K. Zervantonakis, Alisha Schor, Sharon Ong, Devin Neal, Roger D. Kamm, H. Harry Asada

**Affiliations:** 1 Department of Mechanical Engineering, Massachusetts Institute of Technology (MIT), Cambridge, Massachusetts, United States of America; 2 Department of Biological Engineering, Massachusetts Institute of Technology (MIT), Cambridge, Massachusetts, United States of America; 3 BioSym Interdisciplinary Research Group, Singapore-MIT Alliance for Research and Technology (SMART), Singapore, Singapore; Université de Technologie de Compiègne, France

## Abstract

We demonstrate ensemble three-dimensional cell cultures and quantitative analysis of angiogenic growth from uniform endothelial monolayers. Our approach combines two key elements: a micro-fluidic assay that enables parallelized angiogenic growth instances subject to common extracellular conditions, and an automated image acquisition and processing scheme enabling high-throughput, unbiased quantification of angiogenic growth. Because of the increased throughput of the assay in comparison to existing three-dimensional morphogenic assays, statistical properties of angiogenic growth can be reliably estimated. We used the assay to evaluate the combined effects of vascular endothelial growth factor (VEGF) and the signaling lipid sphingoshine-1-phosphate (S1P). Our results show the importance of S1P in amplifying the angiogenic response in the presence of VEGF gradients. Furthermore, the application of S1P with VEGF gradients resulted in angiogenic sprouts with higher aspect ratio than S1P with background levels of VEGF, despite reduced total migratory activity. This implies a synergistic effect between the growth factors in promoting angiogenic activity. Finally, the variance in the computed angiogenic metrics (as measured by ensemble standard deviation) was found to increase linearly with the ensemble mean. This finding is consistent with stochastic agent-based mathematical models of angiogenesis that represent angiogenic growth as a series of independent stochastic cell-level decisions.

## Introduction

The use of microfluidic cell cultures to characterize the response of multi-cellular systems *in vitro* has gained traction in the experimental study of a variety of biological processes, including angiogenesis [1], [2], [3], [4], [5], neuronal growth and development [6], [7], [8], [9], immune cell responses [10] and micro-tissue cultures [11] among many others [12]. Research objectives were largely focused on investigating the effects of various cellular input cues on the cellular response of interest. Input cues include extracellular concentrations and gradients of biochemical growth factors, matrix mechanical properties, flow induced shear stress, and electrical and mechanical stimulation [13]. Cellular responses of interest include cell migration, phenotypic transitions, signaling responses and ensuing morphogenesis. The resulting cue-response relationships are immensely important for elucidating the underlying mechanisms of the biological processes, as well as providing the response characterizations needed for tissue engineering, therapeutic and drug screening applications [14].

Angiogenesis, the biological process of creating new blood vessels from existing ones, is an exemplary case where understanding the roles of multiple exogenous inputs, and differentiating their affects from endogenous factors, is critically important. Understanding the mechanisms of healthy vascularization as well as associated disease states has clear implications on the development of novel cancer therapies [15], [16], drug screening [17], as well as vascular tissue engineering [18], [19], [20].

The formation of three-dimensional lumen-like structures from a uniform monolayer of endothelial cells is a widely used *in vitro* model for experimentally and computationally studying angiogenesis [21], [22]. Angiogenic growth is known to depend on a wide array of soluble biochemical cues, which influence the phenotypic and migratory state of the endothelial cells via autocrine, paracrine and endocrine feedback. These biochemical cues include: i) growth factors such as vascular endothelial growth factors (VEGF) [23], [24], angiopoietins (ANG1) and (ANG2) [25], fibroblast growth factors (α-FGF, β-FGF) [26], platelet derived growth factors (PDGF) [27], ii) chemokines such as PF4 [28], iii) matrix metalloproteinases (MMPs) [29], [30], and iv) signaling lipids such as sphigosine-1-phospate (S1P) [31], [32], [33] among others. In conjunction with mechanical signaling factors [34], the presence of the aforementioned biochemical cues in the extra-cellular domain elicit angiogenic responses such as tip cell differentiation and coordinated migration, leading to the formation of sprouts, branches and capillary lumens, and eventually functional capillary networks [23], [35].

Multiple approaches have been implemented to quantitatively characterize the morphological effects of these biochemical factors on capillary formation. A variety of *in vivo* and *in vitro* angiogenesis assays [35] as well as mathematical models [22], [36], [37], [38], [39] have been implemented to assess their effects on the sprouting response of the endothelial monolayer, and to explain the underlying mechanisms. While three-dimensional microfluidic assays for angiogenic sprouting have been demonstrated [1], [3], [4], [40], there are challenges to increasing their experimental throughput in order to attain ensemble observations necessary for reliable statistical analysis. These challenges include difficulties in attaining device-to-device fabrication consistency and uniformity of cell culture conditions applied to all instances in the ensemble, which consequently result in difficulty in obtaining reliable quantitative estimates of the angiogenic response. In general, statistical analysis of three-dimensional angiogenic responses has been challenging because assay conditions could be uncertain or imprecisely applied, and also because limited observational throughput reduces the ability to characterize a full range of angiogenic distributions.

It is our observation that the throughput of a cell-culture assay is typically inversely proportional to its complexity. For example, single cell assays, measured using flow cytometry, can yield millions of data points [41]. Three-dimensional multi-cellular cluster assays (which are markedly more complex than single cell assays due to cell-cell interactions) can result in hundreds of parallel observations [11], [42]. In contrast, assays that form three-dimensional growth patterns and morphological structures (such as the formation of angiogenic structures), generally allowed for only a handful of observations [3]. Despite increased complexity in such morphology-forming assays, unraveling the role of exogenous micro-environmental factors versus endogenous factors is of importance for treating angiogenic defects, as different drugs and therapeutic strategies may be required depending on each situation which would require increased throughput. A highly controllable *in vitro* experimental platform yielding ensemble observations of three-dimensional angiogenic growth would enable delineation of these effects.

Additionally, increasing the throughput of angiogenic studies can provide insights regarding their statistical properties which cannot be attained from just a few experimental realizations. In studies where ensemble observations are available, not only can averages of the responses be estimated, but also their variances, and possibly their distributions can be characterized [43]. Estimating the variance of the response distribution enables i) better design of experiments to characterize the effects of various input cues on the biological process, ii) understanding and decomposing endogenous and exogenous factors contributing to response and its variance, and iii) potentially identifying cell culture strategies that enable similar expected average outcomes while minimizing resultant variances.

In this work, we develop and demonstrate a new *in vitro* microfluidic assay for characterizing and analyzing ensemble, three-dimensional angiogenic growth. Our approach takes two complementary paths: First, we developed a three-dimensional microfluidic assay enabling an order of magnitude increase in the number of angiogenic observations available from existing platforms [1]. This is attained by increasing the number of parallelized cell cultures under common extracellular conditions. Multiple parallel observations enable better estimates of the distributions, as well as mean and variance statistics of angiogenic growth. Second, through automated image processing techniques, we quantified the angiogenic growth of endothelial cells for the various extracellular test conditions. Our key message is that by combining the ensemble microfluidic cell culture platform with automated image processing, higher throughput quantitative characterizations of multi-cellular responses became feasible, which allow for identifying distributions that govern the angiogenic process. We demonstrate assay functionality by implementing this approach to evaluate endothelial monolayer response to two potent pro-angiogenic growth factors; namely the vascular endothelial growth factor (VEGF-165) and sphingosine-1-phosphate (S1P).

## Materials and Methods

### Microfluidic Device Design

**Figure 1:**
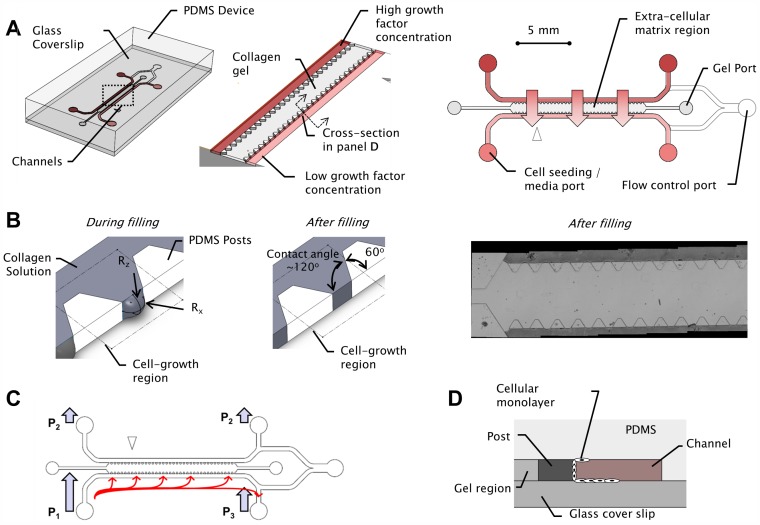
Device concept and layout. A) Overall microfluidic device layout. B) The devices contain an array of trapezoidal posts that cage collagen gel solution into well-defined regions with uniform surface interface. During collagen filling, the gel solution-air interface curvature sustains transient filling pressures.The posts are chosen to have an angle of 60°, supplementary to the contact angle of the liquid collagen and treated PDMS surface (found to be 120°). Post spacing is 100–125 microns, and width of gel region is 1.3 mm. C) To enable binding of cells to the gel region, the hydrostatic pressure in the input ports was managed allow for directional flow of the cells along the length of the device, coupled with interstitial flow that biases the cells against the gel region. Consequently cells bind to the collagen region, and form the basis for the monolayer that develops in the ensuing 24 hours. D) Cross section (defined in panel B) illustrating cellular monolayer forms to confluence after growth of adherent seeded cells.

The microfluidic tissue culture devices used in this study extended the approach described in [1], [2], [3] to incorporate an increased number of cell growth regions, yielding multiple observation instances on a single chip. The devices (Figure 1A) consist of 2 media channels engulfing an extended, central region containing the extracellular gel matrix. By varying the composition of the cellular growth media in the channels, growth factor concentrations and gradients across the gel region are established to stimulate cellular responses. Observations are attained via three-dimensional confocal imaging of the gel region through the supporting glass cover slip. The gel is injected manually in liquid form via pipetting, and allowed to thermally cross-link before culture media and cells are introduced. Cells are seeded in solution (Figure 1C) and cultured to form a monolayer (Figure 1D) that mimics a blood vessel wall, with growth factor gradients applied orthogonal to the monolayer.

Two objectives were sought in the design of the device with respect to gel containment in the central region: i) extending the length of the gel region to enable the formation of a longer monolayer, thereby increasing the number of cell growth regions and their associated observations, and ii) achieving a uniform gel-fluidic channel interface since non-uniformities would result in aberrations in the extra-cellular conditions and non-uniform cell seeding. These two objectives are achieved via post design (Figure 1B). To enable uniform gel-fluidic channel interface, trapezoidal shapes were chosen for the posts. The post angle (60 degrees) was chosen to supplement the contact angle of the PDMS surface (measured to be ∼120 degrees) such that the collagen is flush with the media channels [44].

To extend the length of the gel region, posts were spaced such that the pressure containment capability of the cage was sufficient to withstand filling pressure transients encountered during filling of the gel solution. Ideally, it is desirable to minimize the presence of the posts (in number and in size) since they diminish the usable length of the device. However, since the posts provide the necessary functionality of gel caging, they need to be sufficiently close to maintain sufficient pressure containment capability.

To maximize pressure containment in the gel cage, we analyzed factors driving surface tension at the air-liquid interface. The pressure drop across the air-liquid interface is given by 
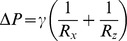
 [45], where Δ*P* is the pressure differential sustained by surface tension, γ is the surface tension coefficient, and *R_x_* and *R_z_* are the radii of curvature of the air-liquid interface in two mutually perpendicular directions (shown in Figure 1B). In conjunction with the contact angle between PDMS and gel solution, *R_x_* is determined by post spacing, whereas *R_z_* is determined by channel height. An interface that is robust to spillage during the filling process is one that can withstand large pressure perturbations Δ*P*. Thus, to maximize Δ*P*, two steps were taken: first, devices were baked during fabrication (as described below) to render PDMS hydrophobic [3], which allowed for the formation of interfaces with smaller radii of curvature. Second, the radius of curvature *R_x_*, was chosen via empirical testing of various post spacings to provide the necessary Δ*P* that allowed the gel to be filled along the length of the device. The radius of curvature *R_z_* was not subject to optimization since it was determined by channel height, and was constrained by the desire to maintain three-dimensionality of the cell culture. Furthermore, the converging geometry of the trapezoidal posts helped stabilize the air-gel interface since it results in narrower radius *R_x_* as more gel is filled in the device. Thus, the geometry provided a stiffening interface that helped arrest perturbations that may have occurred during the manual gel filling process.

### Characterization of Transport Phenomena

**Figure 2:**
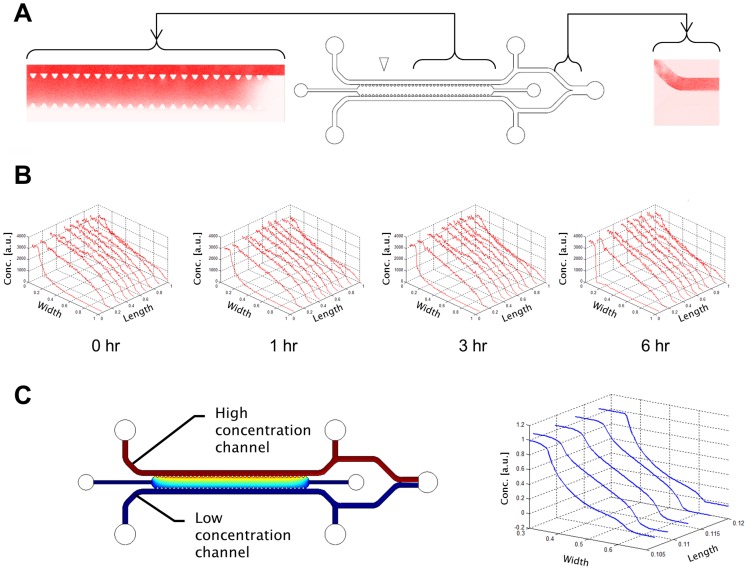
Characterization of device transport characteristics in the absence of monolayers. Characterization was done via Texas Red conjugated 40 kDa Dextran in lieu of VEGF (which has a molecular weight of 38 kDa) A) Gradients are estimated via fluorescent intensity measurements along the entire gel region B) The generation gradients that are stable in time when device is under flow of 2 µL/min. Gradients are shown stable over a 6 hour time period. C) Numeric simulations reveal concentration profiles along the length of the channel, and confirm gradient profiles similar to those observed experimentally.

To characterize transport phenomena in the device, simulations were performed using a diffusion-convection-reaction finite element model implemented in COMSOL (Burlington, MA). The chemoattractant transport model (Figure 2C) corresponded to a 40 kDa growth-factor (such as VEGF165) diffusing inside a 3D collagen type I gel (concentration 2.5 mg/mL). Based on previous experimental characterization [46] we defined a diffusion coefficient *D_GEL_* of 5×10^−11^ m^2^/s for the 40 kDa growth-factor diffusion in the collagen matrix. The diffusion coefficient in the medium-containing channels (*D_MED_*) was defined using the Stokes-Einstein relationship, assuming a 40 kDa globular protein at 37°C yielding a coefficient of 5×10^−11^ m^2^/s, which was similar to the diffusion coefficient in the collagen matrix defined above. A flow-rate of 1 µL/min was defined at each inlet of the chemoattractant and control channels, representing the conditions of the experimental characterization. For the common outlet boundary, downstream the Y-junction, we set the pressure equal to zero and defined the mass flux to be equal to *u*×*C* representing pure convective transport where *u* is the cross-sectional averaged velocity at the exit and *C* is the average concentration. This is supported due to the high Peclet number (*uL/D* ∼50) demonstrating the convection was dominant over diffusion. Due to the device geometry we had to take into account transport in both the axial (along channel length) and lateral (along gel width) directions; hence the Peclet number is calculated as *Pe*  =  *u*×(*h/L)*×*h/D*, where *h* is the channel width and *L* is the total gel region length, *D* the diffusion coefficient and *u* the normal flow velocity. To demonstrate the role of convection in ensuring uniform concentration gradients along the device length, we performed a parametric computational study and found that for flow-rates higher than 0. 1 µl/min the axial variation along the device length was minimal (Figure S1), where *h* is a characteristic length scale (*h* ∼500 um channel width) and *D* the chemoattractant diffusion coefficient. Constant concentration source condition (*C_SOURCE_*) was defined at the inlet of the chemoattractant channel and constant concentration sink (*C_SINK_*) conditions at the inlet of the control channel. Sink concentration boundary conditions were also defined at the boundary of the gel filling port, because of the large volume of gel in the vertical gel filling channel. Growth-factor binding to the collagen type I gel and to the endothelial monolayer was neglected in order to allow for comparison with the concentration gradient characterization experiments where a fluorescent dextran was used. The numerical grid for performing the simulations consisted of approximately 100,000 unstructured finite elements with a quadratic Lagrangian shape function.

### Device Fabrication

The devices were fabricated out of polydimethylsiloxane (PDMS - Dow Corning® Sylgard 184 at a ratio of 10∶1 polymer to cross-linker) using standard soft lithography techniques [47]. Devices were wet autoclaved for 20 minutes followed by a dry autoclave cycle for 20 minutes and baked overnight at 80°C to dry. Devices were plasma bonded to #1.5 glass cover slips (Cell Path) that were pretreated with ethanol. All device channels were then treated with 1 mg/mL poly-D-lysine (PDL) solution (Sigma-Aldrich) for 4 hours to enhance cell and collagen matrix binding to the device material [21], followed by additional baking at 80°C for 24–48 hours to dry and make the devices hydrophobic.

Type I collagen gel solution (BD Biosciences Cat. No. 354236) was prepared at 2.5 mg/mL and pH 7.4, and was pipetted into the devices at low injection pressures to avoid spillage into the main channels. The injection pressures were lower than the upper limit determined by the surface tension cage. Once in place, the collagen solution was allowed to gel for 1 hour in a humidity box via thermal cross-linking. The media channels were then filled with microvascular endothelial growth media (Lonza EGM-2MV Cat. No. CC-3202) to hydrate the gel, and prepare it for endothelial cell adhesion and growth.

### Cell Culture, Staining and Seeding

Human microvascular endothelial cells (hmVECs - Lonza Cat. No. CC-2543) were received at passage 3 and expanded to passage 7 in endothelial growth media (Lonza EGM-2MV Cat. No. CC-3202) via standard mammalian adherent cell culture protocols, and then cryogenically frozen until needed. When cells were needed prior to seeding in a device, a passage 7 vial was thawed and expanded to passage 8. When the cells reached 80–90% confluence, they were stained with the cytosolic stain CellTracker Green CMFDA (Invitrogen Cat No. C7025) at 5 [µM], and with the nuclear stain Hoechst 33342 (Invitrogen Cat. No. H1399) at 0.1 [µM] both using the recommended protocols for CMFDA from Invitrogen. These stain concentrations were found to maintain cell viability as well as sustained image contrast for the duration of the cultures in the devices. After staining, the cells were trypsinized (0.05% Trypsin EDTA, Invitrogen), centrifuged, and then suspended in endothelial growth media to a density of 2.5 million cells/mL. Live staining of the cells enabled confocal imaging before and after biochemical conditions were applied to the gel region.

Cells were seeded through the main flow channels of the devices, while and air gap was maintained at the downstream junction to isolate the two main channels. The hydrostatic pressures across the channels were controlled by pipetting droplets of various sizes for each of the ports (Figure 1C) and were managed to enable net cell convection in the seeding channel along the length of the device. Additionally, the pressures were managed so as to create a slow interstitial flow through the gel that biases the cells towards the gel for adhering on the gel-medium interface (Figure 1C). Care was taken to ensure that cells did not excessively crowd the gel region so as to avoid necrosis.

The cell-seeded devices were allowed to culture for 24 hours before condition was applied to ensure a confluent monolayer. Any devices that did not have a confluent monolayer or exhibited excessive cell buildup on the gel regions were discarded after 24 hours. After the biochemical condition was applied, the channel medium was replaced every 12 hours.

### Image Acquisition

Three dimensional volumetric images of the resulting monolayer and angiogenic structures were acquired via confocal microscopy (Olympus FluoView 1000) using a 20× objective (NA  = 0.75). Four image channels were acquired for each volume: CMDFA fluorescence, Hoechst 33342 fluorescence, reflected light, and transmitted light. Each imaging volume covered one gel growth region. The volumetric Z-stack covered a ∼160 micron range over 18–20 sections per stack.

Images were acquired at two time points: immediately before the conditions was applied (labeled as the 0-hr time point) and 48 hours later (labeled as the 48-hr time point). Due to the large number of cell growth regions in each device and the plurality of devices, time points, and test conditions reported, more than 1000 volumetric confocal images were acquired through the course of the experiments.

### Image Processing

We developed automated image processing methods to quantify the angiogenic response for each of the gel regions in each device. The development of automated methods was necessary to handle the large number of images acquired and to eliminate subjective variance associated with manual processing [48]. Confocal image stacks were processed to extract meaningful angiogenic metrics at the 0-hr and 48-hr time points, thereby enabling differential comparisons to assess the effects of various growth conditions. All image processing algorithms were implemented via custom scripts in Matlab (Mathworks Inc.). Image processing was divided into the following key steps, the outcome of which is illustrated in Figure 4 and S1:

**Figure 3:**
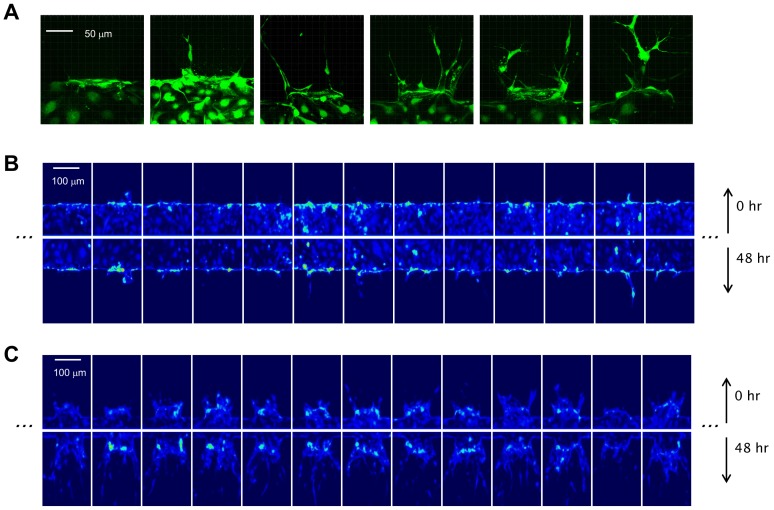
Examples of angiogenic response under various conditions. A) The range of angiogenic responses can be captured in the device from no response to single and multiple sprouts and branches. B) Ensemble observations at basal conditions compared to ensemble observations C) at pro-angiogenic conditions (applied VEGF gradient + S1P). Note that while conditions are applied after the 0 hr images were acquired, variance in initial cellular activity is observed.

#### A. Image registration and gel region detection

By defining boundaries between the gel region and PDMS posts, coordinate systems were defined to enable registrations and comparisons between images of different days. Boundary detection was performed by exploiting the trapezoidal post features visible in each image based on reflectance measurements. Pixel intensity histograms revealed a bimodal distribution of the pixels in the entire imaging domain owing to the difference in character of the pixels inside vs. outside the gel region. The optimal separating boundary was found by numerically searching for the boundary lines that maximize the separation between the two underlying distributions. The search was conducted by casting the separation objective in an optimization framework, and maximizing the difference between the pixel intensity distributions, thereby attaining optimal separation of gel and PDMS.

#### B. Monolayer detection

The objective of this step was to determine the location of the endothelial monolayer. Similar to gel region detection, the problem was also formulated in an optimization framework where the objective was to search for the curve within the gel region that maximizes integrals of monolayer signals in a band around candidate monolayers. Image signals were taken to be a linear combination of cytosolic and transmitted light intensity signal in the image. Candidate lines were parameterized by 4-point cubic splines. The optimizer function searched for and optimized the spline parameters that resulted in a curve that maximized the integral of the cytosolic signal in a band around the candidate curve.

#### C. Computation of angiogenic metrics

**Figure 4:**
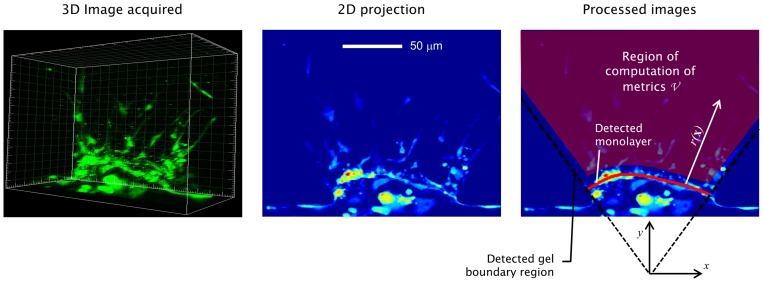
Computation of angiogenic metrics. Input images directly acquired via confocal microscopy are filtered, registered, and were used to determine the boundaries of the gel region, the endothelial monolayer, and coordinate references used for registration and translation between the 0-hr image and the 48-hr condition. These boundaries also define a region of computation of metrics that is slightly offset by 10 microns in order to avoid bias due to the high intensity signals around the monolayer (due to cellular aggregation). Details of image processing steps are described in the Supporting Materials.

Because of the complexity of the three-dimensional sprouting morphologies (as shown in Figure 4), multi-faceted metrics are required to capture the evolution of angiogenic growth. Evaluating metrics based on angiogenic features, such as sprouts, branches, lumens and anastomoses through automated means was practically infeasible due to the complexity of the resulting morphologies and the associated computational costs. Therefore, our objective was to adopt integrative metrics that are i) relevant to the underlying biological phenomena, ii) feasible for implementation on a large scale, and iii) free from human intervention.

Angiogenic metrics were computed using two signals: i) the cytosolic signal *s_cyto_*(**x**) which was based on image intensity of the CMFDA stain, where **x** is the three-dimensional position vector, and ii) the nuclear signal *s_hoechst_* (**x**) based on image intensity of the Hoechst stain. Images were standardized by applying a two-dimensional median filter to minimize noise content, and were normalized by conversion to gray-scale. Low background noise content was identified by fitting Gaussian distributions to pixel intensity histograms, and was correspondingly eliminated.

The metrics were evaluated on a region bounded by the gel-PDMS post interface and the identified cell monolayers. This region, labeled *V* in Figure 4, encompassed most of the angiogenic activity, and defined the domain of integration for the metrics defined below:

- The total cell cytosolic integral and the total nuclear integral invading the gel region above the monolayer. These are computed according to 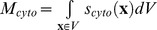 and 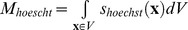 respectively, where *V* is the region of integration previously determined. The outcome of this computation is a scalar quantity that indicates aggregate cellular growth in the gel regions resulting from migration and proliferation from within the monolayer.
- The first moment of the cytosolic integral and nuclear integral, computed according to 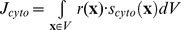 or 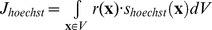, where *r*(**x**) is distance measured normal to the detected monolayer (as shown in Figure 4). This moment therefore represents the degree of penetration of the cells, based on their cytosolic signals, into the gel region past the monolayer.
- The aspect ratio is computed according to 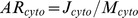 and 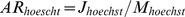. A higher aspect ratio indicates higher directionality of cell penetration into the gel region past the monolayer in the direction of chemoattractants.

The above metrics quantify the overall sprouting activity and the directed migration, delineating the two basic features of angiogenic sprouting. Note that both metrics are evaluated by computing integrals over a growth region. As such they are computationally stable, having low sensitivity to signal noise, outliers, and diverse shape and size of sprouts.

### Angiogenic Test Conditions

Angiogenic test conditions were chosen to sample from a range of VEGF and S1P concentrations, gradients, and combinations thereof. The test conditions are summarized in Table 1, and were applied to the devices after the 0-hr images were acquired. These conditions were replenished every 12 hours, and maintained for 48 hours until the 48-hr images were acquired.

**Table 1:** Test conditions.

Conditionlabel	Condition at monolayerchannel	Condition at oppositechannel	Nominal average conditionacross gel region	Nominal gradient acrossgel region
Control	Basal media (*)	Basal media (*)	Basal media (*)	None
VEGF Grad.	20 ng/mL [VEGF]	40 ng/mL [VEGF]	30 ng/mL [VEGF]	20 ng/mL [VEGF]/channel
VEGF + S1P	20 ng/mL [VEGF]	20 ng/mL [VEGF] +250 nM [S1P]	20 ng/mL [VEGF] +125 nM [S1P]	250 nM [S1P]/channel
VEGF Grad. + S1P	20 ng/mL [VEGF]	40 ng/mL [VEGF] +250 nM [S1P]	20 ng/mL [VEGF] +125 nM [S1P]	20 ng/mL [VEGF] +250 nM [S1P]/channel

* Basal media is as (Lonza EGM-2MV Cat. No. CC-3202) excluding additional VEGF from the bullet kit. Concentrations of conditions in this table are defined in excess of basal media content.

## Results and Discussion

### Microfluidic Devices and Characterization

The device design is shown in Figure 1A. Each device has 37 cell growth regions defined by 2 trapezoidal posts that are spaced at 100 microns apart at their nearest points. Other variations of the device have up to 80 cell growth regions, though only the devices with 37 growth regions were used for cell culture in this study. The post base angles were chosen at 60 degrees to be approximately supplementary to the contact angle between the gel solution and the PDMS surfaces, resulting in a consistently uniform gel-channel interface flush with the posts.

To identify optimal PDMS post geometry for gel containment, characterization devices were designed and fabricated to explore the effects of various geometric parameters (Supplementary Material). These characterization devices considered the effects of post shape, spacing, angles and gel channel width. A post spacing of 100–125 µm was required to ensure robust filling along the length of the device (>15 mm). The gel filling approach described herein is inherently limited to small areas of cell culture growth, albeit a large number of regions. While the distance between posts is sufficient for micro vascular sprouting demonstrations, this gel culture approach may not be sufficient for other applications requiring larger monolayer areas.

To validate growth factor transport dynamics, we used Texas Red-conjugated Dextran (40 kDa) as an analog for biological growth factors such as VEGF-165 (38 kDa). By imaging the fluorescence intensity, diffusion profiles are attained (Figure 2A & B). These characterizations were conducted when flow was drawn through the device at 1 µL/min via a perfusion pump (Harvard Apparatus). Under flow conditions, the device developed nearly linear gradients that can be sustained for extended durations (>6 hours duration of measurements).

While the concentration profiles are close to linear, numerical simulations show that they deviate from linearity due to variation in the collagen matrix cross-section area in the lateral direction (perpendicular to the channel axis). This change is due to the trapezoidal shape of the posts which were necessary for ensuring surface-tension mediated gel caging. Furthermore, the concentration profiles in the outermost gel regions nearest to the gel filling ports were affected by the large mass of gel at the ports that acted as a sink. This resulted in differences in the concentration gradients in the first and last 2 to 3 gel growth regions in the device, as compared to the other gel regions.

### Computational Characterization of Device Transport

The chemoattractant concentration field at steady state in the microfluidic device is shown in Figure 2C. Due to the high Peclet number, the concentration along the microfluidic channel remained nearly constant with the axial variations being small compared to the concentration difference between the two channels, allowing for the establishment of similar concentration gradients across the different gel filling regions along the length of the device. The plotted concentration profiles were spaced 4 mm (10 gel regions) apart. We note here that because the gel filling port acting as a concentration sink, the concentration gradient is steeper in the cell growth regions closest to the gel filling ports, which is consistent with Dextran transport characterization.

The ability to perform detailed parametric studies and accurate prediction of the concentration gradients by combining simulation and experimental characterization are important tools for quantifying the chemoattractant gradient, as sensed by the endothelial cells and influencing their angiogenic potential.

### Tissue Culture and in vitro Angiogenic Response Formation

Cell viability was demonstrated by the formation of a wide range of angiogenic constructs. Figure 3A shows Maximum Intensity Projections (MIPs) of the 3D growth patterns into the gel growth region. Resultant structures exhibited a wide range of responses from single and multiple sprouts, to lumens and branches. Ensemble observations were acquired revealing the range of responses due to the application of various conditions and formed the basis for statistical analyses. A subset of such ensemble conditions is shown in Figures 3B and 3C.

### Statistics of Angiogenic Response

**Figure 5:**
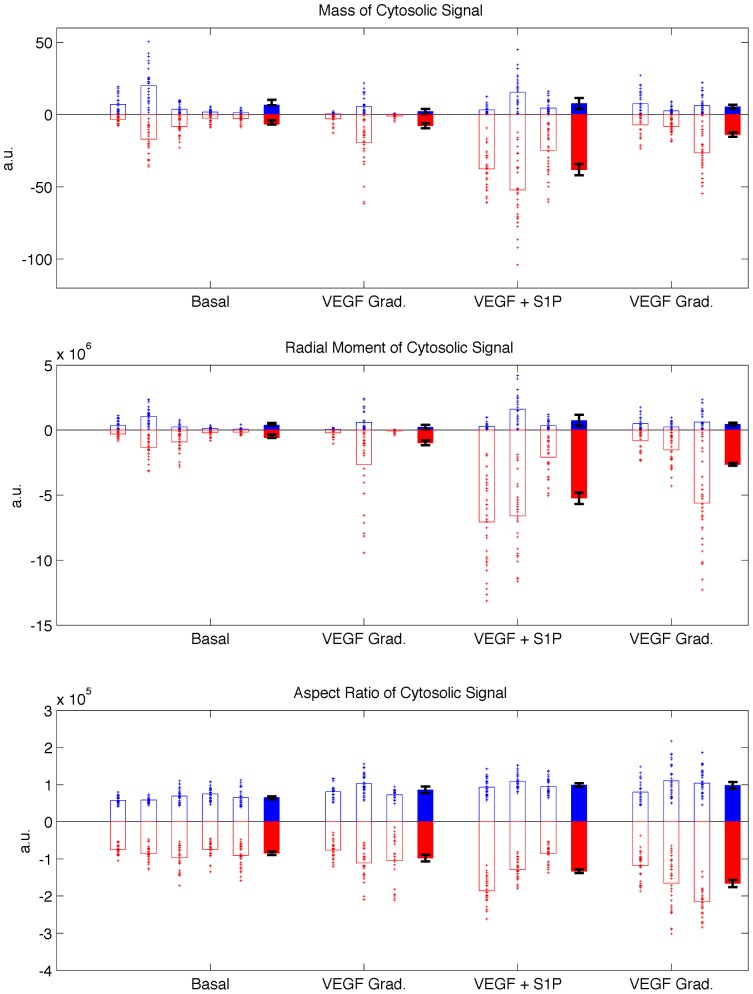
Summary of angiogenic growth metrics. Three measures are shown: A) integral of cytosolic signal *M_cyto_*, B) moment of cytosolic signal J*_cyto_* and C) aspect ratio *AR_cyto_*. Measures at 0 hr (pre-condition) are indicated in blue, and measures at 48 hr are indicated in red (with sign reversed to emphasis asymmetry in growth metrics). Open bars represent the means of each device, with the corresponding markers indicated individual measures over each region. Solid bars represent condition means, with error bars represent +/−1 S.E.

Figure 5 summarizes the angiogenic response metrics computed for 14 devices under the 4 different conditions described in Table 1. The response metrics shown are for cytosolic integral *M_cyto_* invading the gel region (Figure 5A), the moment *J_cyto_* (Figure 5B), and the corresponding aspect ratio *AR_cyto_* (Figure 5C).

Because cells were cultured using two hMVEC batches (both passage 8), control cultures at basal conditions (without any additional VEGF or S1P) were prepared for each batch to normalize for baseline angiogenic activity. In Figure 5, the angiogenic metrics computed at the 0-hr conditions are plotted in the positive sense (blue), whereas angiogenic metrics computed after the condition was applied (48-hr) are plotted in the negative sense (red). Therefore, symmetric plots indicate that no change has occurred due to the application of the conditions. Figure 5 indicates responses of individual cell-growth regions, device averages and conditions averages, and shows the variance within each device.

**Figure 6:**
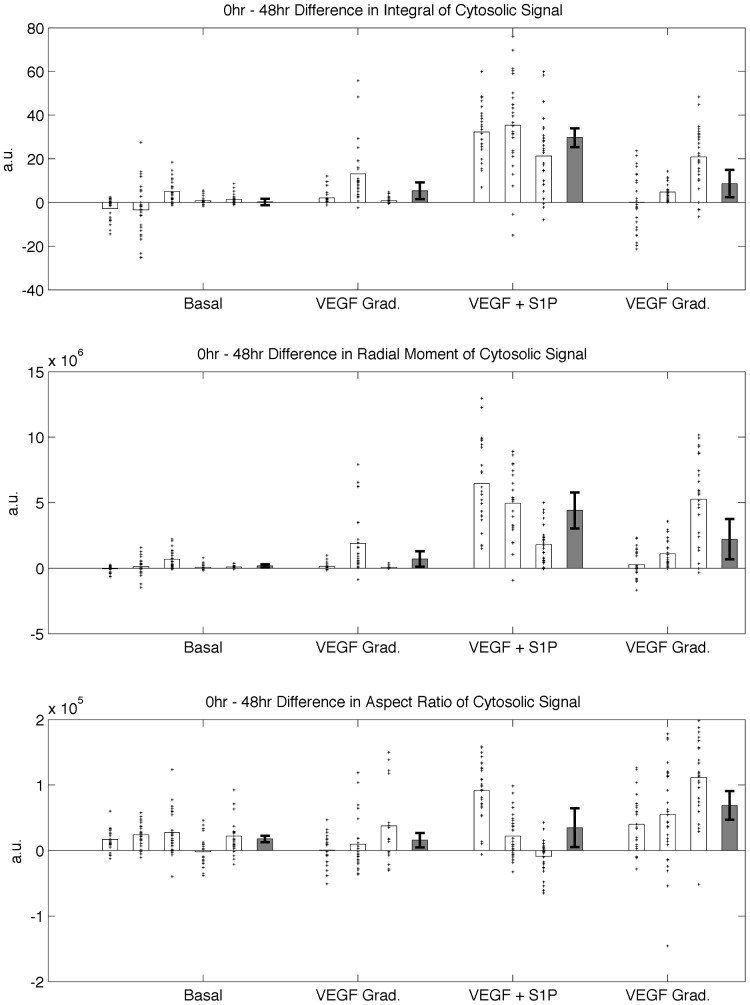
Differential measures of angiogenic growth metrics. Three measures are shown representing 0 hr–48 hr change in: A) integral of cytosolic signal *M_cyto_*, B) moment of cytosolic signal J*_cyto_* and C) aspect ratio *AR_cyto_*. Open bars represent the means of each device, with the corresponding markers indicated individual measures over each region. Solid bars represent condition means, with error bars represent +/−1 S.E. Measures of statistical significance are computed using Student’s *t*-test and computed for each condition in relation to its corresponding basal culture. Markers indicate: (* *p*<0.1), (**p<0.05) and (***p<0.005).

To eliminate the effects of the inherent baseline activity in each device prior to the application of conditions, differential metrics are computed (by subtracting the response at 48-hr from 0-hr) and are plotted in Figure 6 in a manner similar to Figure 5. Statistical significance was tested between the average of each condition (solid bars) and its corresponding control.

### Effects of VEGF and S1P

Figure 6 describes the effects of exogenous biochemical inputs on the ensemble statistics of angiogenic growth, and illustrates the inherently different effects of the growth factors considered on these statistics. When applied in combination with VEGF, S1P significantly enhances the angiogenic response compared to the condition with VEGF gradients alone. This is presumably due to the role of S1P in enhancing the cells’ migratory response. This trend is captured in all computed metrics using both cytosolic and nuclear signals.

In the presence of S1P, a basal level of VEGF leads to increased migration into the gel growth region than over a gradient of VEGF. However, the VEGF gradient + S1P condition exhibited significantly greater directionality in the response evident in the aspect ratio measurements. This suggests the synergistic effects of both growth factors on affecting the growth and directionality of the angiogenic sprouts. This also emphasizes the need for multi-faceted metrics that capture different characteristics of the growth process.

The plots of Figure 5 and Figure 6 were generated based on cytosolic signals. The trends therein did not change significantly when the statistics were generated based on nuclear signals (see Figures S4, S5 and S6).

### Response Variance within a Device: Intrinsic Variance

**Figure 7:**
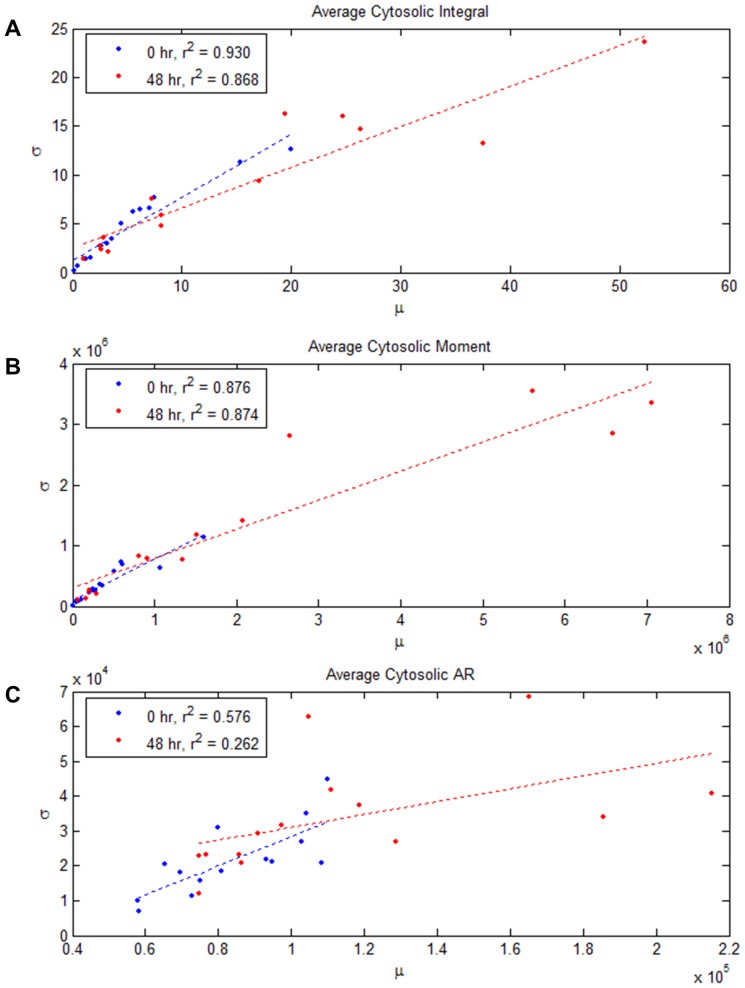
The variance in angiogenic response metrics (based on cytosolic signals) in an ensemble is linearly proportional to the ensemble mean response. The linear relationship was found irrespective of the growth metric used, and relative slopes between 0 hr and 48 hr measures were maintained.

Analyzing sources of variance in observed angiogenic activity is an important consideration in assessing assay repeatability and standard errors of observed growth metrics. The variability of the measured responses in Figures 5 and 6 can be attributed to intrinsic factors that are due to the inherently stochastic cellular response, and extrinsic factors due to the device condition applied (such as the difference in conditions applied across devices, differences in gel cross-linking, and in the baseline activity levels of the different cell batches). We explored the effects of each as follows. To investigate the character of intrinsic variance, and the relationship between device variance and the mean angiogenic response within a device, we plotted the standard deviation of each of the three metrics against the mean response in Figure 7 (see also Supplementary Materials for similar plots based on nuclear signals). The plots summarize the responses for all devices across all conditions considered. Regression fits indicate a strong linear correlation (0.87< r^2^) between the standard deviation and mean responses for both the integral and moment metrics. Linear correlations were less evident in the aspect ratio measurement because it is a ratio between the two aforementioned quantities. This linear relationship between response standard deviation and mean value suggests that multiplicative noise models can be incorporated in stochastic angiogenic models.

Connections can be made between the linear mean-variance response relationship and mathematical models of angiogenesis. Several statistical processes, such as random walks with drift or gamma distributions [49], exhibit the property of having variance proportional to the mean. These processes arise from the summation of a number of more elementary independent processes (for example, exponentially distributed processes in the case of the gamma distribution). Agent-based mathematical models treat angiogenesis responses as an accumulation of independent cell-level decisions [22], [50], and therefore have the inherent structures that can predict the aforementioned mean-variance relationship. Similarly, experimental investigations of cellular migration [51], [52] have shown that cellular migration follows an exponential distribution, whereas their average velocities follow a gamma distribution, which is consistent with our observations.

### Variance Among Devices: Extrinsic Variance

The variance of the mean response of devices sharing common conditions is captured by the standard error bars in Figures 5 and 6. These indicate an inherent variability between devices even when similar extracellular conditions are applied. This variance may be attributed to irregularities in the inherently random cell seeding protocol, which may result in monolayers that are initially more confluent than others, thereby biasing future response. This variance also indicates that a single device, though capable of generating ensemble observations given a set of extracellular conditions, these observations are also conditioned on device specific factors. Therefore, a plurality of devices is still needed to fully characterize the response population.

### Advantages of Ensemble Analysis

The advantage of an ensemble tissue culture device with a large number of cell growth regions can be quantified in terms of the standard errors of process estimates within each device, and among different devices. It can be shown (see Text S1) that the for a total number of *k* cell growth regions distributed among *m* devices, the optimal number of cell growth regions in each ensemble device that minimizes total measurement standard error is given by 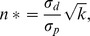 where 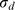 is the device standard deviation, 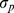 is the variance of device means under the same applied conditions. Consequently, the optimal number of devices would be 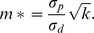 Thus the ratio 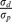 determines the relative distribution of cell growth regions in a device. Large relative device variance 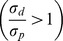 implies the need for ensemble devices with more cell growth regions, whereas large relative processes variance 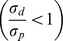 implies the need for more devices and fewer growth regions in each device. Based on the acquired data, this ratio ranged between 1.2 and 12, with 7 being a typical value for aspect ratio measures, which supports the need for ensemble measurements.

### Other Potential Uses of the Device and Ensemble Characterizations

Other potential uses for this device and methodology include cancer transmigration assays, 3D cell migration and control experiments, as well as co-culture of multiple cell types such as endothelial and smooth muscle cells. Ensemble characterizations of cellular response means and variances can give useful insights these processes as well as to the effects of potential interventions and controls.

### Conclusions

Ensemble characterizations of complex multi-cellular angiogenic responses are necessary to distinguish between different sources of variance: those that are due to inherent cellular stochasticity versus *in vitro* assay heterogeneity. Towards this goal, we developed and demonstrated a microfluidic assay, and accompanying image analysis and angiogenic metrics computation methods to characterize the statistics of angiogenic responses under the influence of VEGF and S1P, two well-known pro-angiogenic biochemical factors. Results confirm the importance of S1P in amplifying the angiogenic response in the presence of VEGF gradients. Furthermore, the application of S1P with VEGF gradients resulted in angiogenic sprouts with higher aspect ratio than S1P with background levels of VEGF, despite reduced total migratory activity. Finally, intrinsic variance in the computed angiogenic metrics (as measured by ensemble standard deviation) was found to increase linearly with the mean response. This is compatible with models describing angiogenesis in terms of agent-based stochastic responses. This linear relationship mean-variance relationship was found to hold irrespective of the particular metrics used or the growth conditions applied to the device.

## Supporting Information

Figure S1 **The change in concentration profiles along the channel width depends on channel flow rate (q).** These results are shown based on the numerical model described in the main text.
(TIFF)

Figure S2 **The optimal boundary separating the gel region from the rest of the device is the one the maximizes the separation between pixel intensity distributions.** The sepration is measured by maximizing the difference in means between the two candidate distributions. A) Image of the reflectance channel. B) Pixel intensity distribution for the entire image. C) Pixel intensity distributions inside vs. outside the gel region. D) Corresponding cytosolic image.
(TIFF)

Figure S3 **Overview of key steps to image processing include image registration, monolayer detection, and calculation of endothelial penetration parameters.** Figure shows summary of processing of confocal images. A) Input images are reflectance (which contrasts gel and PDMS structures), cytosolic stain, nuclear stain and transmitted light. All images are 2D median-filtered and background noise is threshold. B) Reflectance images are used to define the straight edges of the gel region. Candidate edges are evaluated on the basis of maximizing the difference of pixel distribution between candidate gel and regions and exterior. Best fit is found via direct optimization of separating boundary parameters. C) A summation of transmitted light and cytosolic signals is used to fit the monolayer. Monolayers are fit to a four point cubic spline. The fits are performed by maximizing the integral of the cytosolic and reflectance signals over a band surrounding each candidate splines. Best fits are attained by maximizing over optimal spline parameters. The monolayer boundary and the gel region boundary define the range of integration in the image. D) The cytosolic and nuclear images are masked by the region of integration. The resulting image is used to calculate the metrics described in the text.
(TIFF)

Figure S4 **Summary of angiogenic growth metrics.** Three measures are shown: A) integral of nuclear signal *M_hoescht_*, B) moment of nuclear signal J*_hoescht_* and C) aspect ratio *AR_hoescht_*. Measures at 0 hr (pre-condition) are indicated in blue, and measures at 48 hr are indicated in red (with sign reversed to emphasis asymmetry in growth metrics). Open bars represent the means of each device, with the corresponding markers indicated individual measures over each region. Solid bars represent condition means, with error bars represent +/−1 S.E. Bracketed conditions were cultured using cells from the same batch.
(TIFF)

Figure S5 **Differential measures of angiogenic growth metrics.** Three measures are shown representing 0 hr-48 hr change in: A) integral of nucelar signal *M_hoescht_*, B) moment of nuclear signal J*_hoescht_* and C) aspect ratio *AR_hoescht_*. Open bars represent the means of each device, with the corresponding markers indicated individual measures over each region. Solid bars represent condition means, with error bars represent +/−1 S.E. Bracketed conditions were cultured using cells from the same batch. Measures of statistical significance are computed using Student’s *t*-test and computed for each condition in relation to its corresponding basal culture. Markers indicate: (* *p*<0.1).
(TIFF)

Figure S6 **The variance in angiogenic response metrics (based on nuclear signals) in an ensemble is linearly proportional to the ensemble mean response.** The linear relationship was found irrespective of the growth metric used, and relative slopes between 0-hr and 48-hr measures were maintained.
(TIFF)

Text S1 **Supporting information text.**
(DOCX)
