# Supplementary figures and images for: Ensemble Analysis of Angiogenic Growth in Three-Dimensional Microfluidic Cell Cultures
Source: PLoS One. 2012 May 25;7(5):e37333. doi: 10.1371/journal.pone.0037333 (PMC3360734; doi:10.1371/journal.pone.0037333)

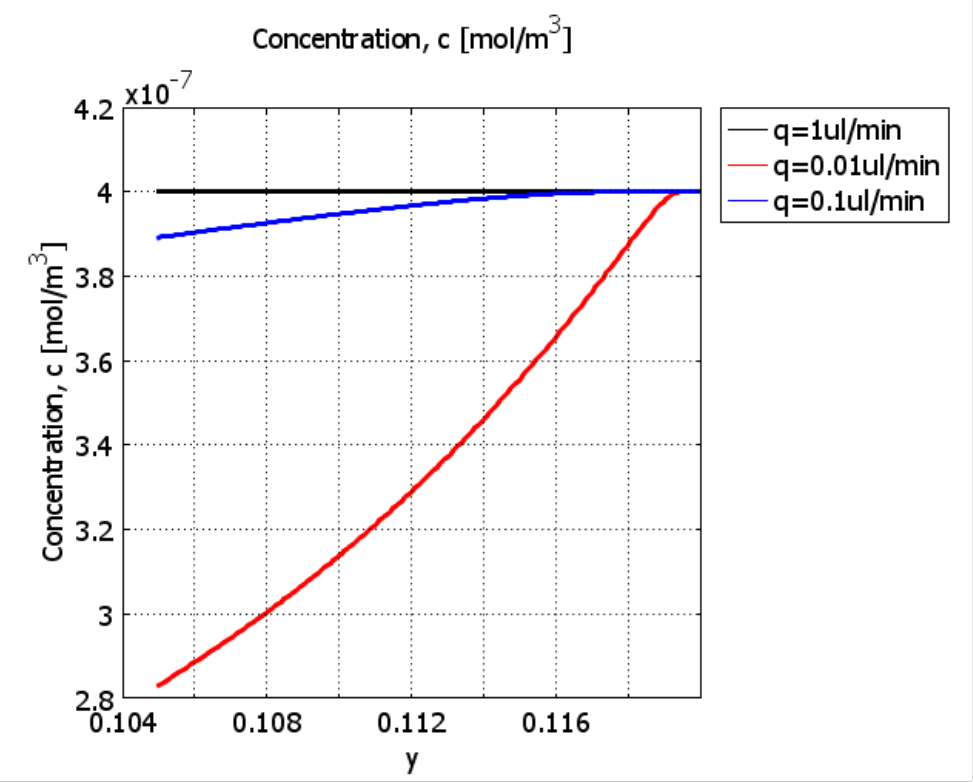

Supplement: Figure S1 — The change in concentration profiles along the channel width depends on channel flow rate (q). These results are shown based on the numerical model described in the main text. (TIFF) [file pone.0037333.s001.tiff]

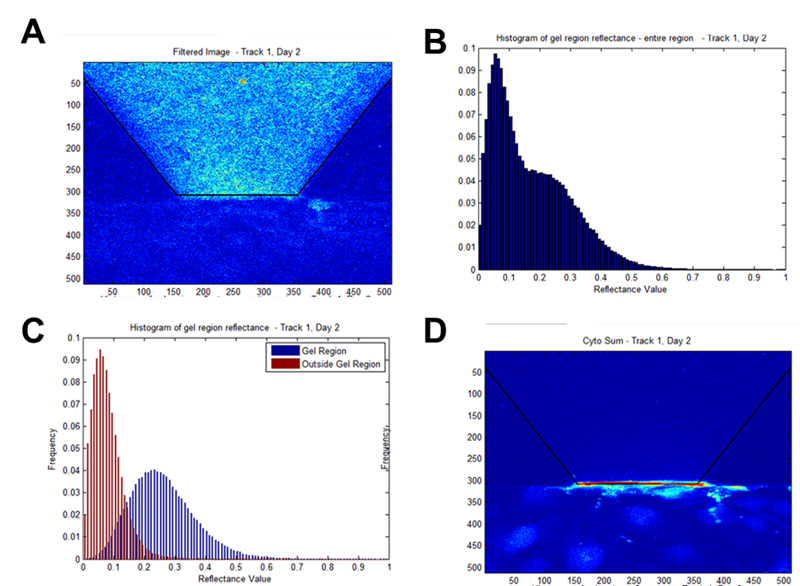

Supplement: Figure S2 — The optimal boundary separating the gel region from the rest of the device is the one the maximizes the separation between pixel intensity distributions. The sepration is measured by maximizing the difference in means between the two candidate distributions. A) Image of the reflectance channel. B) Pixel intensity distribution for the entire image. C) Pixel intensity distributions inside vs. outside the gel region. D) Corresponding cytosolic image. (TIFF) [file pone.0037333.s002.tiff]

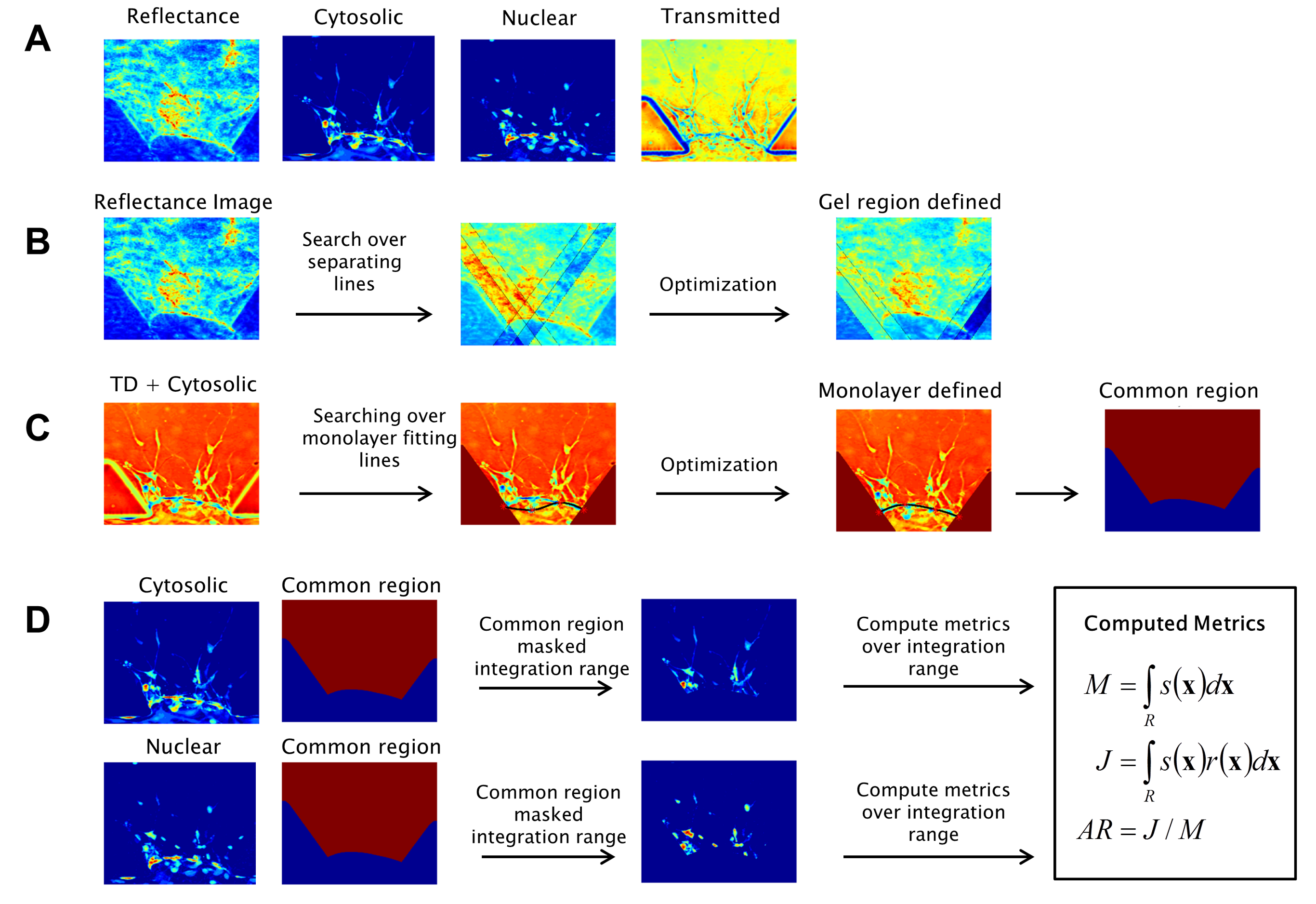

Supplement: Figure S3 — Overview of key steps to image processing include image registration, monolayer detection, and calculation of endothelial penetration parameters. Figure shows summary of processing of confocal images. A) Input images are reflectance (which contrasts gel and PDMS structures), cytosolic stain, nuclear stain and transmitted light. All images are 2D median-filtered and background noise is threshold. B) Reflectance images are used to define the straight edges of the gel region. Candidate edges are evaluated on the basis of maximizing the difference of pixel distribution between candidate gel and regions and exterior. Best fit is found via direct optimization of separating boundary parameters. C) A summation of transmitted light and cytosolic signals is used to fit the monolayer. Monolayers are fit to a four point cubic spline. The fits are performed by maximizing the integral of the cytosolic and reflectance signals over a band surrounding each candidate splines. Best fits are attained by maximizing over optimal spline parameters. The monolayer boundary and the gel region boundary define the range of integration in the image. D) The cytosolic and nuclear images are masked by the region of integration. The resulting image is used to calculate the metrics described in the text. (TIFF) [file pone.0037333.s003.tiff]

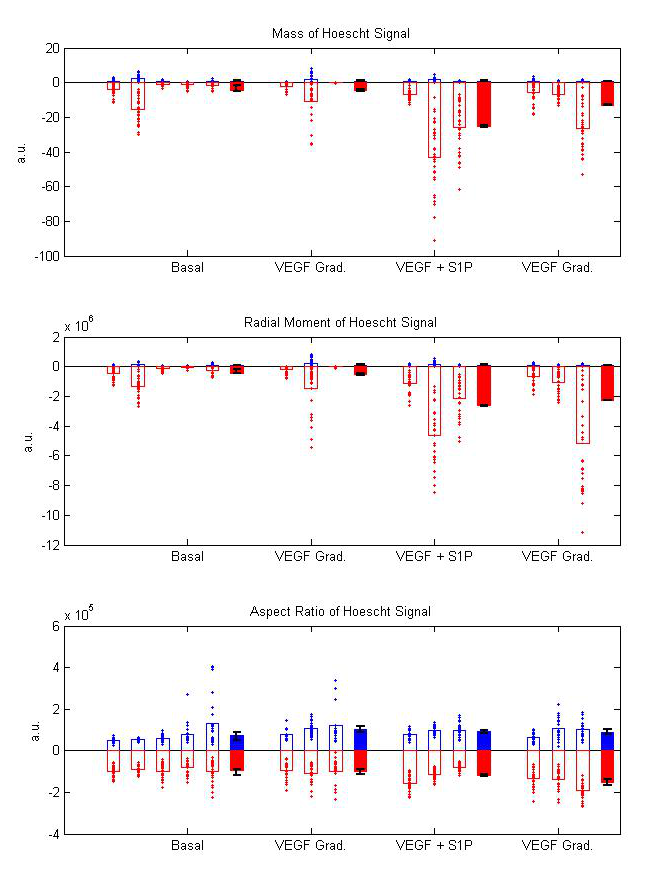

Supplement: Figure S4 — Summary of angiogenic growth metrics. Three measures are shown: A) integral of nuclear signal Mhoescht, B) moment of nuclear signal Jhoescht and C) aspect ratio ARhoescht. Measures at 0 hr (pre-condition) are indicated in blue, and measures at 48 hr are indicated in red (with sign reversed to emphasis asymmetry in growth metrics). Open bars represent the means of each device, with the corresponding markers indicated individual measures over each region. Solid bars represent condition means, with error bars represent +/−1 S.E. Bracketed conditions were cultured using cells from the same batch. (TIFF) [file pone.0037333.s004.tiff]

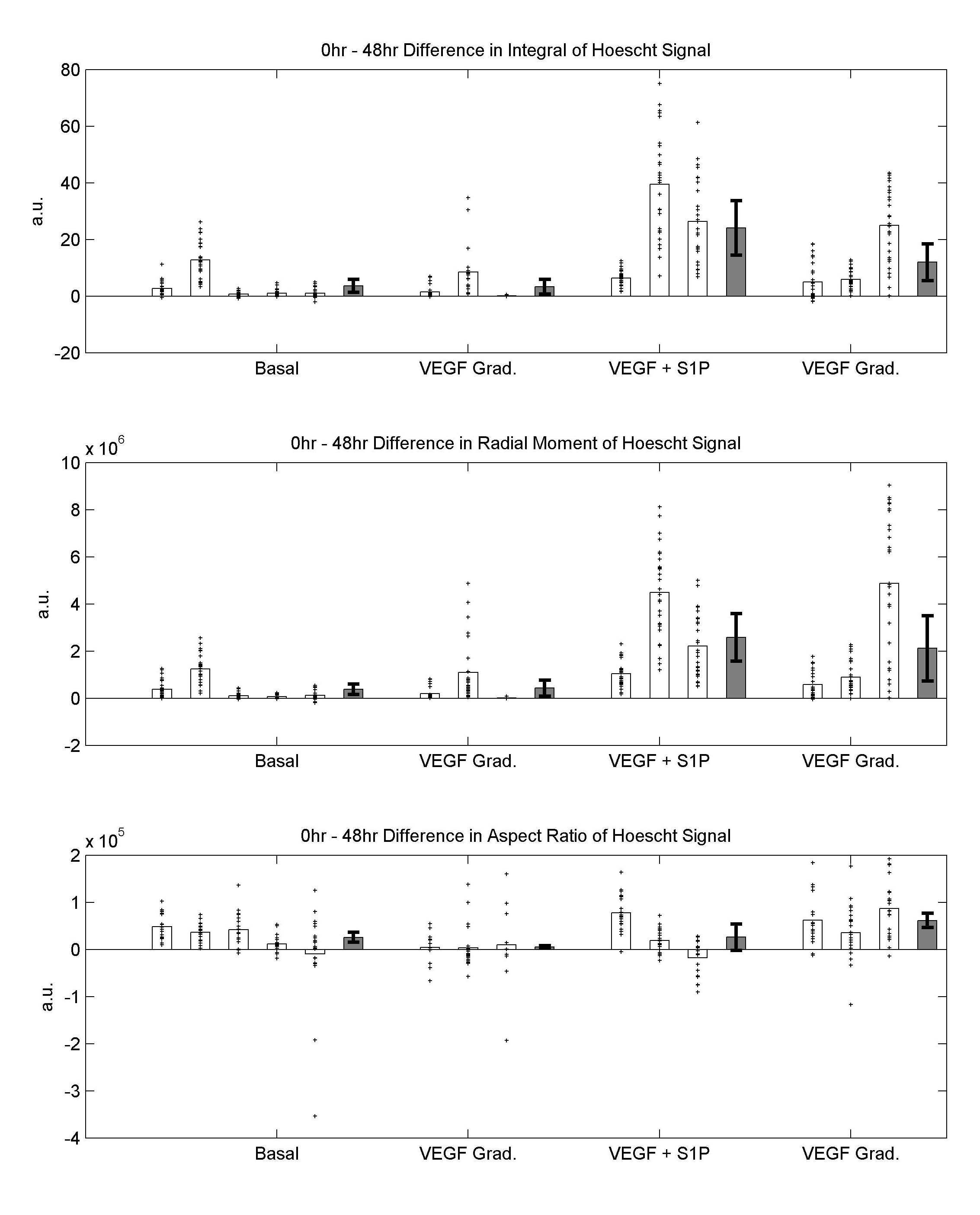

Supplement: Figure S5 — Differential measures of angiogenic growth metrics. Three measures are shown representing 0 hr-48 hr change in: A) integral of nucelar signal Mhoescht, B) moment of nuclear signal Jhoescht and C) aspect ratio ARhoescht. Open bars represent the means of each device, with the corresponding markers indicated individual measures over each region. Solid bars represent condition means, with error bars represent +/−1 S.E. Bracketed conditions were cultured using cells from the same batch. Measures of statistical significance are computed using Student’s t-test and computed for each condition in relation to its corresponding basal culture. Markers indicate: (* p<0.1). (TIFF) [file pone.0037333.s005.tiff]

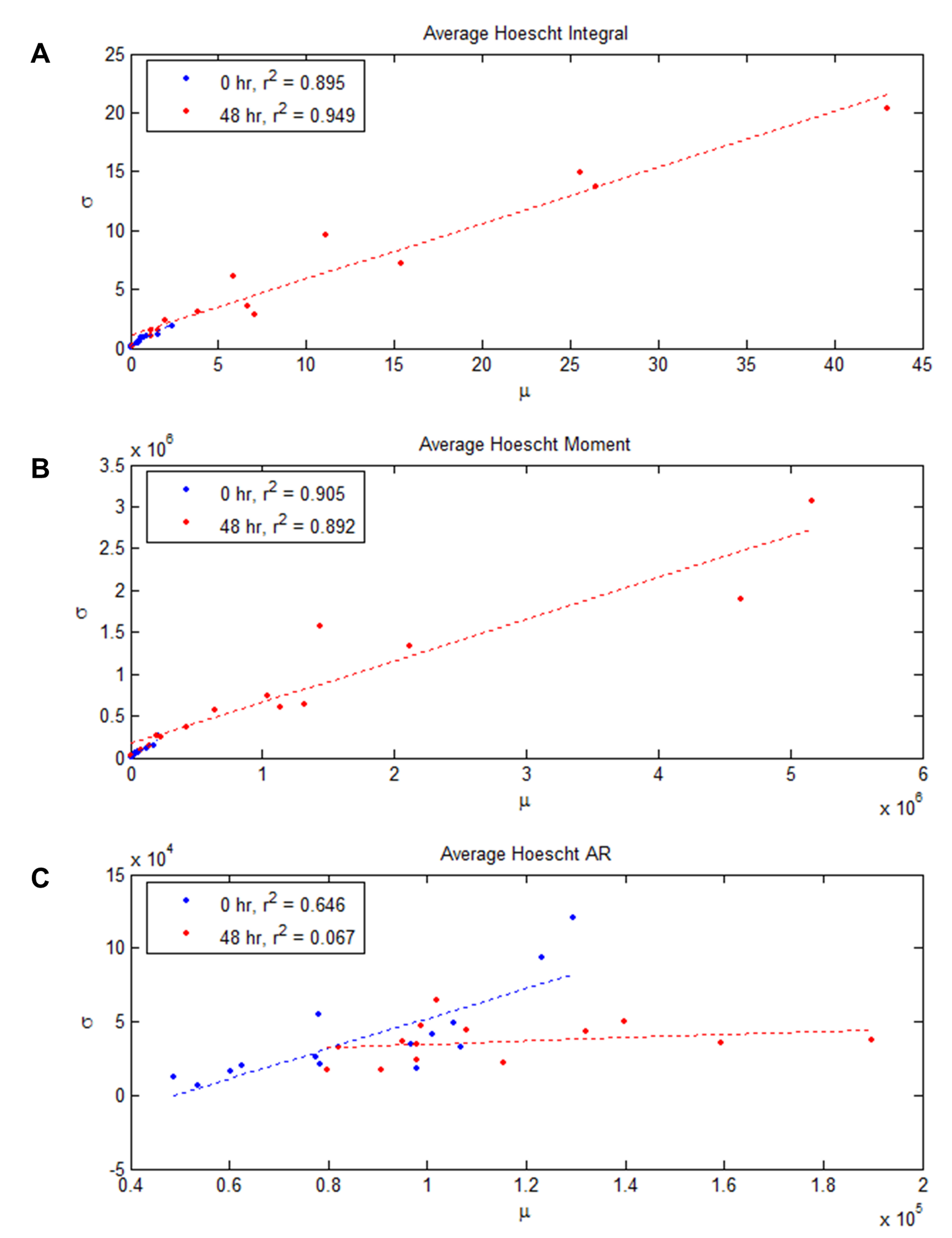

Supplement: Figure S6 — The variance in angiogenic response metrics (based on nuclear signals) in an ensemble is linearly proportional to the ensemble mean response. The linear relationship was found irrespective of the growth metric used, and relative slopes between 0-hr and 48-hr measures were maintained. (TIFF) [file pone.0037333.s006.tiff]
