# Supplementary material for: Ensemble Analysis of Angiogenic Growth in Three-Dimensional Microfluidic Cell Cultures
Source: PLoS One. 2012 May 25;7(5):e37333. doi: 10.1371/journal.pone.0037333 (PMC3360734; doi:10.1371/journal.pone.0037333)
Supplement: Text S1 — Supporting information text. (DOCX) [file pone.0037333.s007.docx]

# Supporting Information

## Image processing

This section adds detail to the image processing steps followed to compute the angiogenic metrics described in the text. The procedure was described in general terms in the main text, and an overview of the main steps is outlined in Figure S2 and its caption.

Three-dimensional images were acquired over four channels. The four channels were: i) cytosolic signal using the CMFDA stain, ii) nuclear signal using the Hoechst stain, iii) reflectance, and iv) transmitted light. Volumetric images of each channel are acquired over 18-20 planes, resulting in a voxel size of 0.62x0.62x7 microns, where 7um is the distance between the imaging planes.

Because the final statistics of angiogenic measures are inherently differential, the measures must be made over identical volumes of cell growth to render comparisons meaningful. Since the positioning of the microfluidic devices at the start and end of each experiment changes the imaging field, we relied on the trapezoidal features of the device posts to enable image registration in the x-y directions, and the detection of the coverslip surface for registration in the z direction. To register the image in the x-y directions, we searched for the separating boundaries that maximize the difference in means of the pixel intensity distributions of the reflectance channel between the “inside” and “outside” regions of a candidate separating profile. An example of this separation is shown in Figure S2. Once the images are registered, their common intersection is taken as the volume for computation of angiogenic metrics as described in the text. This volume is indicated as the “common integration range” in Figure S3.

To account for any potential degradation of image intensity over time (due to stain decay), images were converted to gray scale, resulting in normalized pixel intensity distribution values between 0 and 1, where 1 corresponds to the maximum pixel intensity within the common integration range. We then computed the normalized pixel intensity distributions, and observed that they are multimodal distributions, with clear indication of background sub-distributions vs. image sub-distributions. Based this decomposition, the thresholds for background rejection were determined and the rest of the image pixels were retained.

Finding the optimal boundary for seprating the gel region from the rest of the device, as well as finding the optimal monolayer curve fit were both cast as optimization problems. These problems were solved numerically for each individual images using Matlabs *fminsearch* function.

## Statistics of angiogenic metrics based on nuclear measures

The statistics of growth metrics displayed in Figures 5, 6 and 7 are based on cytosolic signals. These statistics are replicated in Figures S4, S5 and S6 based on nuclear signals. The plots follow the same trends and patterns of the main text figures, indicating the changing the form of the signal would not have influenced the conclusions.

**Determination of optimal ensemble size**

The standard error in single device observations is given by ${S.E.}_{d}^{2}=\frac{\sigma_{d}^{2}}{n}$, where $\sigma_{d}$ is the device standard deviation of all observations from a given device having $n$ gel growth regions. Similarly ${S.E.}_{p}^{2}=\frac{\sigma_{p}^{2}}{m}$, where $\sigma_{p}$ is the process standard deviation, and $m$ is the number of devices used to sample the angiogenic process. Assuming independence, the total standard error of the observations is given by
${S.E.}_{total}^{2}={S.E.}_{d}^{2}+{S.E.}_{p}^{2}= \frac{\sigma_{d}^{2}}{n} + \frac{\sigma_{p}^{2}}{m}$. To obtain the most accurate measurements, it is desirable to minimize ${S.E.}_{total}^{2}$ by increasing the $m$ and $n$ as much as is experimentally feasible. Typically, however, there is a constraint limiting the total number of cell growth regions that can be cultured to $k$ regions. This constraint is expressed by $k=mn$. Therefore, the total standard error can be written as

$${S.E.}_{total}^{2}= \frac{\sigma_{d}^{2}}{n} + \frac{\sigma_{p}^{2}}{m}=\frac{\sigma_{d}^{2}}{n} + \frac{\sigma_{p}^{2}m}{k}$$

Minimizing this expression with respect to $n$ results in the following optimal parameters used in the main text:

$$n^{*}=\frac{\sigma_{d}}{\sigma_{p}}\sqrt{k}$$

$$m^{*}=\frac{\sigma_{p}}{\sigma_{d}}\sqrt{k}$$

These values can therefore be used to design optimal experimental parameters for a limited number of observations given device standard deviation and process standard deviation.
